# Toe-to-room temperature gradient correlates with tissue perfusion and predicts outcome in selected critically ill patients with severe infections

**DOI:** 10.1186/s13613-016-0164-2

**Published:** 2016-07-11

**Authors:** Simon Bourcier, Claire Pichereau, Pierre-Yves Boelle, Safaa Nemlaghi, Vincent Dubée, Gabriel Lejour, Jean-Luc Baudel, Arnaud Galbois, Jean-Rémi Lavillegrand, Naïke Bigé, Jalel Tahiri, Guillaume Leblanc, Eric Maury, Bertrand Guidet, Hafid Ait-Oufella

**Affiliations:** Service de réanimation médicale, Hôpital Saint-Antoine, Assistance Publique-Hôpitaux de Paris (AP-HP), 184 rue du Faubourg Saint-Antoine, 75571 Paris Cedex 12, France; Université Pierre et Marie Curie-Paris 6, Paris, France; Service de santé publique, AP-HP, Hôpital Saint-Antoine, 75571 Paris Cedex 12, France; Service de Réanimation polyvalente Quincy-sous-Sénart, Générale de Santé, Hôpital Privé Claude Galien, Quincy-Sous-Sénart, France; Department of Anesthesiology and Critical Care Medicine, Faculty of Medicine, Université Laval, Québec, QC Canada; Inserm U1136, 75012 Paris, France; Inserm U970, Centre de recherche cardiovasculaire de Paris (PARCC), Paris, France

**Keywords:** Sepsis, Shock, Microcirculation, Outcome, Temperature gradient

## Abstract

**Background:**

Microcirculatory disorders leading to tissue hypoperfusion play a central role in the pathophysiology of organ failure in severe sepsis and septic shock. As microcirculatory disorders have been identified as strong predictive factors of unfavourable outcome, there is a need to develop accurate parameters at the bedside to evaluate tissue perfusion. We evaluated whether different body temperature gradients could relate to sepsis severity and could predict outcome in critically ill patients with severe sepsis and septic shock.

**Method:**

We conducted a prospective observational study in a tertiary teaching hospital in France. During a 10-month period, all consecutive adult patients with severe sepsis or septic shock who required ICU admission were included. Six hours after initial resuscitation (H6), we recorded the hemodynamic parameters and four temperature gradients: central-to-toe, central-to-knee, toe-to-room and knee-to-room.

**Results:**

We evaluated 40 patients with severe sepsis (40/103, 39 %) and 63 patients with septic shock (63/103, 61 %). In patients with septic shock, central-to-toe temperature gradient was significantly higher (12.5 [9.2; 13.8] vs 6.9 [3.4; 12.0] °C, *P* < 0.001) and toe-to-room temperature gradient significantly lower (1.2 [−0.3; 5.2] vs 6.0 [0.6; 9.5] °C, *P* < 0.001) than in patients with severe sepsis. Overall ICU mortality rate due to multiple organ failure (MOF) was 21 %. After initial resuscitation, toe-to-room temperature gradient was significantly lower in patients dead from MOF than in the survivors (−0.2 [−1.1; +1.3] °C vs +3.9 [+0.5; +7.2] °C, *P* < 0.001) and the difference in gradients increased during the first 24 h. Furthermore, toe-to-room temperature gradient was related to tissue perfusion parameters such as arterial lactate level (*r* = −0.54, *P* < 0.0001), urine output (*r* = 0.37, *P* = 0.0002), knee capillary refill time (*r* = −0.42, *P* < 0.0001) and mottling score (*P* = 0.001).

**Conclusions:**

Toe-to-room temperature gradient reflects tissue perfusion at the bedside and is a strong prognosis factor in critically ill patients with severe infections.

**Electronic supplementary material:**

The online version of this article (doi:10.1186/s13613-016-0164-2) contains supplementary material, which is available to authorized users.

## Background

During the last decade, fundamental research and intravital microscopy in human helped to understand the unique pathophysiology of severe sepsis and septic shock compared with other types of shock. Those researches highlighted two key points. First, it demonstrated the heterogeneity and the complexity of microcirculatory disorders in response to bacterial invasion, such as modifications in vasomotor tone, activation of the coagulation cascade and increased platelet–leucocyte interactions [1]. Second, it demonstrated the discrepancy between global hemodynamic parameters (such as arterial blood pressure) and microcirculatory blood flow [2, 3]. Studies also have reported a decrease in microcirculatory blood flow in patients with septic shock [4], even when global hemodynamic parameters seemed adequate [5]. Moreover, microcirculatory alterations have been considered to be a stronger determinant of outcome than global hemodynamic parameters [6]. These accumulating pathophysiological and epidemiological evidences explained why the last conference of European experts excluded arterial blood pressure from the definition of shock but stressed the identification of microvascular alterations through the detection of tissue hypoperfusion [7].

As microcirculatory disorders have been identified as strong predictive factors of unfavourable outcome, there is a need to develop accurate parameters at the bedside to evaluate tissue perfusion [8]. We previously reported that the mottling extension [9, 10] and the capillary refill time (CRT) [11] are strongly related to other tissue perfusion parameters (i.e. arterial lactate level and urine output) and strongly predictive of ICU mortality in patients with septic shock. However, their use is limited in patients with dark skin. On the contrary, central temperature and temperature gradients between different body compartments can be measured in all patients no matter the colour of their skin and have been proposed as parameters to evaluate peripheral perfusion in critically ill patients. In a cohort of patients who required ICU admission for trauma, sepsis and surgical complications, Kaplan et al. [12] reported that patients with cold extremities, evaluated in a subjective manner, had a higher arterial lactate level and a lower SVO_2_ than patients with warm extremities. Fifty years ago, Joly et al. [13] studied toe-to-room temperature gradient in a mixed ICU population (patients with cardiogenic, hypovolemic or septic shock and shock due to poisoning). They reported a significantly lower toe-to-room temperature gradient in ICU non-survivors than in ICU survivors. However, temperature gradient has never been specifically and objectively quantified during septic shock, a distributive acute circulatory failure where microcirculatory alterations and endothelial dysregulation predominate. The aim of this study was to evaluate the relationship between temperature gradients and sepsis severity and whether temperature gradients could predict outcome in critically ill patients with severe infections.

## Methods

We conducted a prospective observational study in an 18-bed ICU at a tertiary teaching hospital in France. During a 10-month period, we included all adult patients (≥18 years of age) who required ICU admission for a severe sepsis or septic shock (according to the 2001 SCCM/ESICM/ACCP/ATS/SIS International Sepsis Definitions Conference) from any causes [14]. Patients could be admitted from the emergency department or the medical wards. Patients with hypothermia (defined as central temperature <35 °C) were excluded. Patients with severe sepsis were included at ICU admission, and patients with septic shock were included when vasopressors were required (within 24 h of admission). In septic shock patients, vasopressor initiation was defined as H0.

### Protocol for the management of patients

Management of patients with severe sepsis and septic shock was guided by our local protocol, adapted from international guidelines [15]. In patients with septic shock, intravenous volume expansion was provided to achieve predefined endpoints: pulse pressure variation <13 % [16], no response to passive leg raising [17] or no respiratory variations of the inferior vena cava diameter [18]. Norepinephrine was used in a stepwise manner to achieve predefined endpoints: mean arterial pressure (MAP) ≥65 mmHg and urine output ≥0.5 mL/kg/h. All patients were investigated with transthoracic echocardiography (Vivid 7 Dimension’06, GE, Healthcare^®^). When a cardiac dysfunction (left ejection fraction <30 % by Simpson’s biplane methodology) was identified, an inotropic therapy was introduced and/or epinephrine replaced norepinephrine. Ventilation support was provided when needed. If required, patients were sedated with propofol and/or midazolam and analgesia provided with sufentanil. Use of low doses hydrocortisone (200 mg/day) was considered when there was persistence of vasopressors requirement despite a perceived adequate intravascular volume. Glycemic control and venous thrombosis prophylaxis were provided according to Surviving Sepsis Campaign Guidelines [15].

### Data collection

The following general characteristics of the patients were recorded: age, sex, previous chronic illnesses, severity of illness evaluated by the Sequential Organ Failure Assessment score (SOFA score) within 6 h of inclusion [19] and Simplified Acute Physiologic Score II (SAPS II) [20], primary site of infection, mode of ventilation and vasopressors use. We collected global hemodynamic parameters (MAP, heart rate (HR) and cardiac index) and microcirculatory dysfunction and organ perfusion parameters (arterial lactate level, urine output, mottling score and knee CRT) at study inclusion and at 6, 12 and 24 h following inclusion. Evaluation of mottling score and knee CRT was not done in patients with dark skin.

Toe and knee skin temperature was measured using a skin temperature sensor (STS-400 Level1^®^, Smiths Medical, Rockland, MA, USA) applied at patient inclusion and remained for the next 24 h. Central body temperature was measured with an electronic rectal thermometer (SureTemp^®^ Plus 692 Electronic Thermometer, Welch Allyn^®^), and room temperature was also recorded. The four temperature gradients (°C) were calculated as follows: central-to-toe (central T–toe T), central-to-knee (central T–knee T), toe-to-room (toe T–room T) and knee-to-room (knee T–room T). Outcome at ICU discharge was recorded by the physician at bedside who was blinded to the temperature gradients. Patients were classified as survivors, dead from multiple organ failure (deaths from MOF group) or dead from secondary complications or do-not-resuscitate orders (DNR) (late deaths group). The MOF deaths group consists in patients who had not recovered from MOF secondary from their severe sepsis or septic shock and died following the bacterial injury/invasion. The late deaths group was characterized by hemodynamic improvement and vasopressors weaning, but these patients ultimately died from secondary complications or do-not-resuscitate orders (DNR).

### Statistical analysis

Patient characteristics were expressed as median (25–75th percentiles) or percentages as appropriate. Differences among groups were assessed using the Kruskal–Wallis test with post hoc Mann–Whitney analysis with adjustment for multiple comparisons. Association analyses were performed using the Chi-squared test. Correlation analyses were performed using the Spearman test. When correlation was significant, a linear regression model was fit to the data. The toe-to-room temperature gradient compared with mottling score was tested using the Kruskal–Wallis test. Toe-to-room temperature gradient was analysed as a function of time using analysis of variance for repeated measures. The model included terms for the outcome (survivors, deaths from MOF and late deaths), the time (continuous) and an interaction term between time and outcome. Receiver operating characteristics (ROC) curves were constructed to compare the accuracy of gradient temperature in the prediction of death from MOF. We used logistic regression to model MOF death as a function of patient’s characteristics. All tests were computed with the R software. Significance was defined as a two-sided *P* < 0.05.

Temperature gradients were recorded blindly by an independent physician who did not participate in patient’s care. The protocol was approved by our institution’s ethical committee *Comité de Protection des Personnes (CPP Saint*-*Louis, Paris, France).*

## Results

During a 10-month period, 103 consecutive adult patients who required ICU admission for severe infections were included in the study (Table 1). The median time between ICU admission and inclusion was 2 [0; 5] h. There were 40 patients with severe sepsis (40/103, 39 %) and 63 patients with septic shock (63/103, 61 %). The most prevalent primary sites of infection were the lungs (43 %) and the abdomen (21 %). Most of the patients had comorbidities such as cirrhosis, malignancy or diabetes. Patients with septic shock had higher SAPS II and SOFA scores and required more frequently support therapies (such as mechanical ventilation) than patients with severe sepsis. All patients with septic shock received vasopressor therapy, but four of them were weaned in the first 6 h after inclusion. The vasopressor mainly used was norepinephrine (at H6, 58/59 patients, median dose 0.5 [0.2; 0.9] µg/kg/min) and one patient received epinephrine (at H6, 0.9 µg/kg/min). Thirteen patients had dark skin leaving 90 patients for the evaluation of mottling score and knee CRT.

**Table 1:** Characteristics of patients

Characteristics of patients at H6	Severe sepsis (*n* = 40)	Septic shock (*n* = 63)	*P* value
N	40	63	–
Age (years), median and IQRs	65 [56; 73]	68 [60; 83]	NS
Male gender, (%)	57	66	NS
*Comorbidities (%)*			
Diabetes	25	20	NS
Cirrhosis	5	21	<0.05
Vascular disease	25	32	NS
Solid malignancies	22	22	NS
Haematologic malignancies	22	13	NS
*Primary site of infection (%)*			
Lung	43	43	NS
Abdomen	25	19	NS
Urinary tract	7	14	NS
Soft tissue	15	13	NS
Other	10	11	NS
SAPS II, median and IQRs	37 [28; 46]	60 [46; 69]	<0.001
Mechanical ventilation (%)	28	75	<0.001
*Norepinephrine*			
n	−	58	−
Dose [μg/kg/min]	−	0.5 [0.2; 0.9]	
*Epinephrine*			
n	−	1	−
Dose [μg/kg/min]	−	0.9	

SAPS II was recorded at H24, Simplified Acute Physiology Score. Data are expressed as number, percentage or median and interquartiles (IQRs)

Of the 63 patients with septic shock, 20 patients (20/63, 32 %) died within the first days from multiple organ failure and shock (deaths from MOF group). Forty-three patients (43/63, 68 %) survived the episode of initial septic shock and were weaned from vasopressors, but 11 of them died later from various causes (11/63, 17 %, late deaths group, Additional file 1: Figure S1). Those patients died from acute myocardial infarction (*n* = 2), stroke (*n* = 1), bleeding (*n* = 2), pulmonary aspiration (*n* = 1), secondary infections (*n* = 2) and following a withdrawal of life-sustaining therapies (*n* = 3). Thirty-three patients were discharged alive from ICU (33/63, 52 %). In the severe sepsis group, two patients developed a shock after 24 h of inclusion and died from multiple organ failure. Six patients died later (28 [18; 44] days) from others causes (Additional file 1: Figure S1).

### Hemodynamic parameters assessment according to sepsis severity

**Fig. 1:**
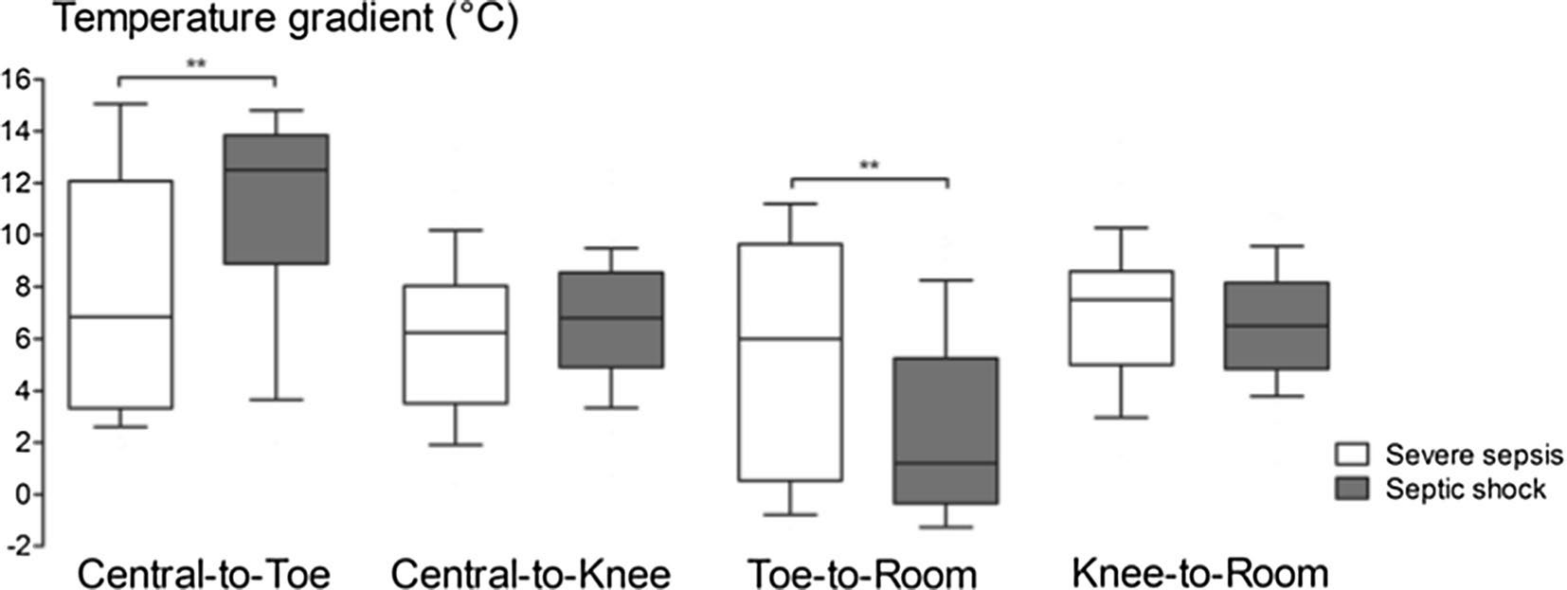
Temperature gradients measured at 6 h in patients with severe sepsis or septic shock. **P < 0.01

Hemodynamic parameters at 6 h according to sepsis severity are presented in Table 2. After initial resuscitation, mean arterial blood pressure and cardiac index were not different between the severe sepsis and the septic shock groups. However, tissue perfusion parameters were significantly different between groups (Table 2). Compared to patients with severe sepsis, patients with septic shock had a significantly lower urine output, higher arterial lactate level, higher knee CRT and a larger mottling score. Central-to-knee and knee-to-room temperature gradients were not different between groups. However, central-to-toe temperature gradient was significantly higher (12.5 [9.2; 13.8] vs 6.9 [3.4; 12.0] °C, *P* < 0.001) and toe-to-room significantly lower (1.2 [−0.3; 5.2] vs 6.0 [0.6; 9.5] °C, *P* < 0.001) in patients with septic shock (Fig. 1).

**Table 2:** Haemodynamic parameters of patients with severe sepsis and septic shock

Haemodynamic parameters at H6	Severe sepsis (*n* = 40)	Septic shock (*n* = 63)	*P* value
N	40	63	–
SOFA score	4 [3; 5]	12 [8; 14]	<0.001
MAP (mmHg)	75 [69; 84]	71 [67; 76]	NS
CI (L/min/m^2^)	2.7 [2.1; 3.4]	2.5 [2.0; 3.0]	NS
Urinary output (mL/kg/h)	1.0 [0.7; 1.4]	0.5 [0.1; 1.2]	<0.001
Lactate level (mmol/L)	1.2 [0.9; 1.9]	2.3 [1.4; 6.0]	<0.001
Index CRT (s)	1.6 [1.0; 2.3]	2.5 [1.7; 4.5]	0.002
Knee CRT (s)	2.2 [1.2; 2.9]	4.2 [2.8; 5.8]	<0.001
*Mottling score (n)* ^*a*^			
0–1	35	35	0.005
2–3	2	12	
4–5	0	6	

Data are expressed as number or median and IQRs

*SOFA* Sequential Organ Failure Assessment, *MAP* mean arterial pressure, *CI* cardiac index, *CRT* capillary refill time

^a^Patients with dark skin were excluded

### Hemodynamic parameters assessment according to ICU outcome

Hemodynamic parameters at H6 according to ICU outcome (survivors, deaths from MOF group and late deaths group) are presented in Table 3. Patients in the survivors and late deaths groups were similar in terms of severity, organ failures and vasopressor use. However, patients in the MOF deaths group were different than patients in the survivors and late deaths groups. Patients who died from an early MOF had a more severe disease as illustrated by a higher SOFA score (14 [12, 15] vs 6 [4, 9], *P* < 0.001), a higher SAPS II (77 [63; 91] vs 43 [33; 54], *P* < 0.001) and a higher need in vasopressors in comparison with survivors (Table 3). Mean arterial blood pressure and cardiac index were not different according to ICU outcome. Regarding tissue perfusion parameters and temperature gradients, there was no difference between the survivors and late deaths groups. However, when compared with survivors, patients in the deaths from MOF group had a significantly lower urine output (0.1 [0; 0.5] vs 1.0 [0.6; 1.4] mL/kg/h, *P* < 0.001), higher arterial lactate level (6.1 [2.7; 14.1] vs 1.5 [0.9; 2.3] mmol/L, *P* < 0.001), higher knee CRT (5.1 [2.9; 9.0] vs 2.8 [1.4; 4.3] s, *P* < 0.001), higher index CRT (4.0 [2.1;5.9] vs 1.8 [1.0; 3.1] s, *P* = 0.002) and larger mottling score (*P* < 0.001).

**Table 3:** Hemodynamic parameters of patients with severe sepsis or septic shock according to ICU outcome

Parameters at H6	Survivors	MOF deaths	Late deaths	*P* value
N	64	17	22	−
Time of death (days)	−	18 [12; 37]	2 [1; 4]	c***
SOFA	6 [4; 9]	8 [5; 11]	14 [12; 15]	a*** *b* ns c***
SAPS II	43 [33; 54]	51 [43; 60]	77 [63; 91]	a*** *b* ns c***
Mechanical ventilation (%)	67	75	90	*a* ns *b* ns *c* ns
Norepinephrine dose (μg/Kg/min)	0.0 [0.0; 0.30]	0.10 [0.0; 0.25]	0.65 [0.50; 3.25]	a*** *b* ns *c* ***
MAP (mmHg)	73 [67; 80]	70 [64; 78]	70 [66; 83]	*a* ns *b* ns *c* ns
CI (L/min/m^2^)	2.6 [2.1; 3.3]	2.4 [1.9; 2.8]	2.7 [1.9; 3.5]	*a* ns *b* ns *c* ns
Urinary output (mL/Kg/h)	1.0 [0.6; 1.4]	0.7 [0.5; 0.8]	0.1 [0; 0.5]	a*** *b* ns c***
Lactate level (mmol/L)	1.5 [0.9; 2.3]	1.5 [1.2; 2.0]	6.1 [2.7; 14.1]	a*** *b* ns c***
*Gradient (°C)*				
Central-to-toe	10.4 [4.6; 12.9]	7.0 [3.7; 12.2]	12.9 [10.5; 14.5]	a** *b* ns c**
Central-to-knee	6.8 [4.6; 8.2]	5.2 [4.2; 7.2]	6.7 [3.9; 9.1]	*a* ns *b* ns *c* ns
*Gradient (°C)*				
Toe-to-room	3.9 [0.5; 7.2]	4.0 [0.5; 9.0]	−0.2 [−1.1; 1.3]	a*** *b* ns c**
Knee-to-room	6.8 [5.1; 8.7]	7.9 [6.4; 8.5]	5.5 [4.1; 7.8]	a* *b* ns c*
Index CRT (s)	1.8 [1.0; 3.1]	2.0 [1.5; 3.3]	4.0 [2.1; 5.9]	a** *b* ns *c* ns
Knee CRT (s)	2.8 [1.4; 4.3]	3.1 [2.2; 4.7]	5.1 [2.9; 9.0]	a*** *b* ns c*
*Mottling score*				
0–1	51	12	7	a***
2–3	5	2	7	*b* ns
4–5	1	1	4	c*

Data are expressed as number, percentage or median and IQRs

*SOFA* Sequential Organ Failure Assessment, *SAPS II* Simplified Acute Physiology Score, *MAP* mean arterial pressure, *CI* cardiac index, *CRT* capillary refill time

Statistical analysis, *a* survivors vs MOF deaths (multi-organ failure), *b* survivors vs late deaths, *c* deaths from MOF vs late deaths

* P < 0.05, ** P < 0.01, *** P < 0.001

Central-to-knee temperature gradient was not statistically different according to the ICU outcome (Table 3). However, central-to-toe temperature gradient was significantly larger and knee-to-room and toe-to-room temperature gradients were significantly lower in the deaths from MOF group in comparison with survivors. The difference was more pronounced for toe-to-room gradient (−0.2 [−1.1; +1.3] for death from MOF group and +3.9 [+0.5; +7.2] °C for survivors, *P* < 0.001). In a multivariable regression, we found that toe-to-room temperature gradient remained associated with death from MOF (OR 0.7 [0.5, 0.9], *P* < 0.001) after adjustment on acute severity, measured by the SOFA score (OR 1.5 [1.2, 1.8], *P* < 0.001), and after adjustment on morbidity, as measured by the presence of malignancy (OR 4.3 [1.1, 16.7], *P* = 0.03) (Table 4).

**Table 4:** Multivariable logistic regression analysis of risk factors measured at H6 for MOF death

	Crude OR (95 % CI)	Adjusted OR (95 % CI)	*P*
*Toe-to-room temperature gradient*			
Per additional degree Celsius	0.7 [0.6, 0.9]	0.7 [0.5, 0.9]	<0.001
*SOFA score*			
Per additional point	1.4 [1.2, 1.6]	1.5 [1.2, 1.8]	<0.001
*Malignancy*			
Yes versus no	2.4 [0.9, 6.3]	4.3 [1.1, 16.7]	0.03

**Fig. 2:**
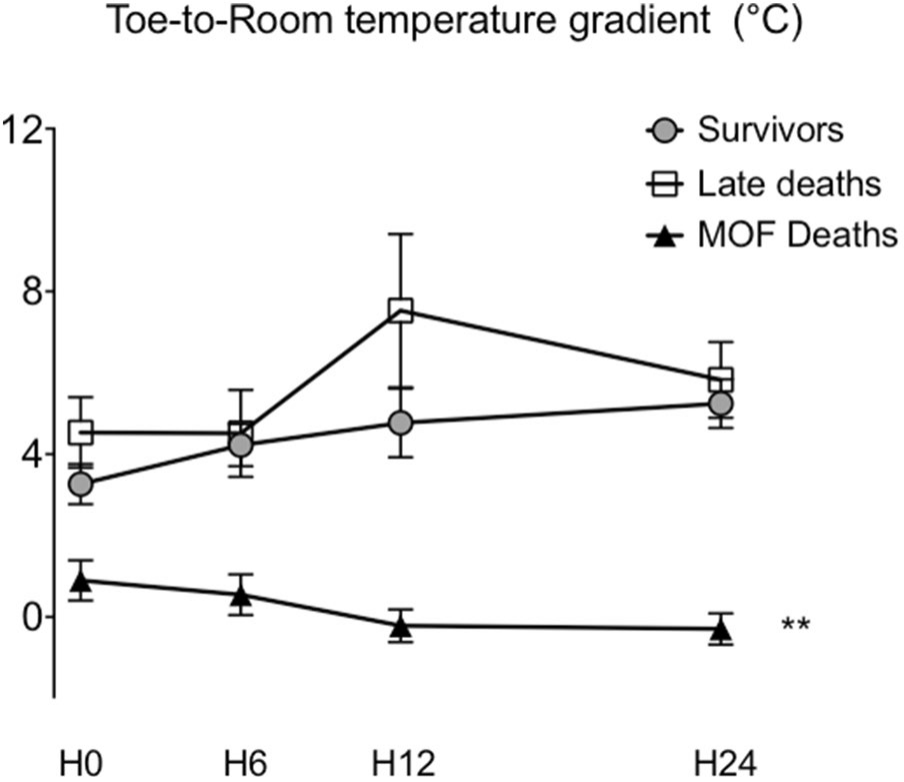
Time course of toe-to-room temperature gradients of pooled severe sepsis/septic shock patients according to ICU outcome. Three groups, survivors, MOF deaths (multiple organ failure) and late deaths. **P < 0.01

In the analysis of variance, the difference in toe-to-room gradient between the three groups was maintained over time, with a gradient decreasing by an average of 0.04 ± 0.05 °C/h in the deaths from MOF group but increasing by 0.08 ± 0.05 °C/h (*P* < 0.03) in the survivors (vs deaths from MOF, *P* = 0.001) (Fig. 2) and increasing by 0.06 ± 0.07 °C/h in the late deaths group (vs deaths from MOF, *P* = 0.006). The toe-to-room temperature gradient was predictive of death due to MOF at H6 with an area under the curve (AUC) of 0.76 [0.65; 0.86]. Predictive value increased over time, at H12 the AUC was 0.83 [0.71; 0.95] and at H24 it reached 0.84 [0.74; 0.94]. At H24, a threshold of toe-to-room temperature gradient of 1.75 °C was predictive of death from MOF with a sensitivity of 75 % (CI 95 %, 53; 98) and a specificity of 75 % (CI 95 %, 62; 85).

**Fig. 3:**
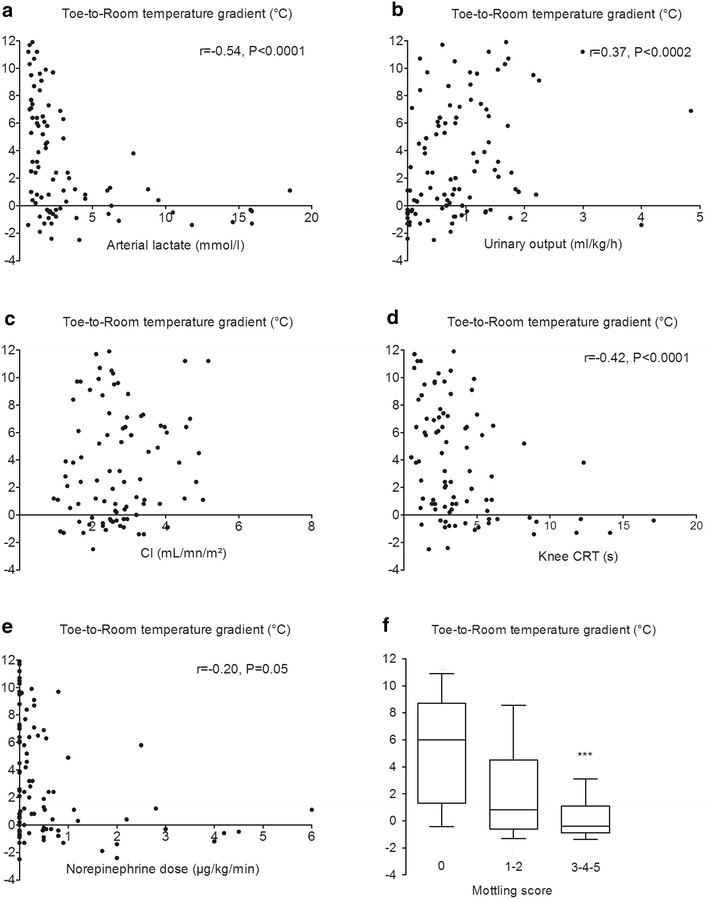
Relationship between toe-to-room temperature gradient and hemodynamic parameters in a pooled analysis of patient with severe sepsis and septic shock at H6; **a** arterial lactate level, **b** urinary output, **c** cardiac index, **d** knee capillary refill time, **e** norepinephrine dose and **f** mottling score. ***P < 0.001

We also analysed correlations between toe-to-room temperature gradient and other hemodynamic variables both measured at H6 after initial resuscitation (Fig. 3) in a pooled analysis of patients with severe sepsis and patients with septic shock. The toe-to-room temperature gradient did not correlate with cardiac index but correlated weakly with vasopressor doses (*r* = −0.20, *P* = 0.05). In contrast, we observed a significant relationship between toe-to-room temperature gradient and tissue perfusion variables such as arterial lactate level (*r* = −0.54, *P* < 0.0001), urine output (*r* = 0.37, *P* = 0.0002), knee CRT (*r* = −0.42, *P* < 0.0001) and mottling score (*P* = 0.001) (Fig. 2). When analysing only patients with septic shock, we found the same correlation between toe-to-room temperature gradient and arterial lactate level, urine output, knee CRT and mottling score. However, the correlation between toe-to-room temperature gradient and vasopressor doses was not significant (data not shown).

## Discussion

In this prospective observational study of 103 critically ill patients with severe infections, central-to-toe and toe-to-room temperature gradients were statistically different between patients with severe sepsis and patients with septic shock. After initial resuscitation, central-to-toe, toe-to-room and knee-to-room temperature gradients were associated with death from MOF. The toe-to-room temperature gradient showed the largest difference between survivors and patients dead from MOF. This difference increased during the first 24 h of ICU admission. Finally, the toe-to-room temperature gradient significantly correlated with tissue perfusion markers such as urine output, arterial lactate level, knee CRT and mottling score.

Temperature gradients are available clinical tools, but their use had not yet been standardized. This is the first study reporting an objective evaluation of temperature gradients in a selected population of patients with severe infections. Kaplan et al. [12] subjectively described in a mixed surgical ICU population that patients with cold extremities had higher arterial lactate levels. Lima et al. [21] proposed a subjective combined approach of tissue perfusion and defined an abnormal peripheral perfusion if the examined extremity had an increase in index CRT or if it was cool to the examiner hands. In another study, patients with abnormal perfusion had a higher forearm-to-fingertip temperature gradient and a higher central-to-toe temperature difference (10 ± 4.1 vs 6.5 ± 3.4 °C, *P* < 0.001). Central-to-toe temperature differences that we observed were in the same range than data reported in the Lima’s study with a median central-to-toe temperature gradient of 12.5 [9.2; 13.8] °C in the septic shock group and 6.9 [3.4; 12.0] °C in patients with severe sepsis. Joly et al. [13] quantified objectively toe-to-room temperature gradient with a skin probe on the ventrum of the great toe and reported a significant lower gradient in non-ICU survivors. However, the authors included a mixed ICU population.

Several clinical studies have highlighted that changes in tissue perfusion parameters following resuscitation were related to prognosis. We reported that survival increased with mottling improvement in patients with septic shock [9]. In a small population of patients with severe sepsis and septic shock, Hernandez et al. recently described that an early recovery of both CRT and central-to-toe temperature gradient at H6 was predictive of lactate normalization at H24 [22]. Here, we found that the variations of toe-to-room temperature gradients were different according to outcome and the cause of death. The temperature gradient decreased in patients dead from MOF during the first 24 h of management, whereas it increased in survivors and patients in the late death group. In non-selected ICU patients with acute circulatory failure, Henning et al. [23] also observed an increase in toe-to-room temperature gradient to more than 4 °C in survivors, whereas this gradient did not reach 3 °C over an interval of 12 h in non-survivors.

We measured skin temperature on the knee because mottling, a prognosis factor during septic shock, is present mainly in this area [24]. In addition, we have recently reported that CRT measured on the knee area was a better prognosis parameter than CRT measured on the finger tip [11]. We found that central-to-knee and knee-to-room temperature gradients were not related to sepsis severity, but knee-to-room temperature gradient was related to ICU outcome.

We analysed the relationship between the temperature gradients and the hemodynamic parameters. The toe-to-room temperature gradient showed the largest difference between survivors and late dead patients. There was no relationship between toe-to-room temperature gradient and cardiac index. In paediatric ICU patients, the association between the core/peripheral temperature gradient and the cardiac index has been reported to be either weak [25] or non-significant [26]. Joly et al. [13] found a significant correlation (*r* = 0.73, *P* < 0.01) between the temperature gradient and cardiac output in a mixed ICU population, but another group did not. Vincent et al. [27] reported a good correlation in patients with cardiogenic shock but not in patients with septic shock. We identified a significant correlation between toe-to-room temperature gradient and tissue perfusion variables such as urine output, arterial lactate level, knee CRT and mottling score. This suggests that room-to-toe temperature gradient reflects more the peripheral tissue perfusion than the global hemodynamic status and also suggests that it could replace mottling score or CRT in patients with dark skin.

We assessed whether confounding factors could affect temperature gradients. We observed a significant but weak correlation between toe-to-room temperature gradient and vasopressor doses in a pooled analysis of patient with severe sepsis and septic shock. However, when analysing only patients with septic shock, this relationship was not significant. The association between MOF-related mortality and toe-to-room gradient temperature measured at H6 was unaffected by stratification on known arterial disease (defined as a previous vascular event, symptomatic or requiring therapeutic intervention) or by stratification on room temperature [28]. It is noteworthy that we applied no exclusion criteria in order to be as close as possible to the “real life”, and to identify a parameter that could be widely used in critically ill patients. We excluded two patients that had low central temperature at admission (33.1 and 34 °C). Some of the patients included suffered cardiovascular disease or cirrhosis, both conditions known to impair vascular reactivity and peripheral perfusion [29]. The heterogeneity of the included population might explain the statistically significant but mild relationship between toe-to-room temperature gradient and tissue perfusion parameters.

Our study is observational, and the next step should be to evaluate whether a therapeutic approach based on clinical evaluation of tissue hypoperfusion including toe-to-room temperature gradient could improve the prognosis of critically ill patients with severe infections. Two recent preliminary studies support this hypothesis. Lima et al. [30] described that nitroglycerin infusion improved forearm-to-fingertip temperature gradient in few patients with acute circulatory failure from 3.3 ± 0.7 to 0.7 ± 0.6 °C (*P* < 0.05). Van Genderen et al. [31], in a proof-of-concept study including 30 septic shock patients, reported that a therapeutic strategy based on peripheral perfusion evaluation, including temperature gradient, led to a trend towards less fluid infusion compared with conventional regimen.

In this study, we investigated the predictive value of temperature gradients, but we did not analyse the underlying mechanisms leading to gradient changes according to outcome. In the context of severe infection, sympathetic activation and endothelial dysfunction could both participate to impairment of distal blood flow and in fine to extremities temperature changes.

## Conclusions

In a prospective observational study of critically ill patients with severe infections, toe-to-room temperature gradient reflected disease severity and correlated with tissue perfusion parameters. Toe-to-room temperature gradient and its variations following resuscitation are independent predictors of mortality due to MOF in patients with septic shock.

## Additional file

10.1186/s13613-016-0164-2 Flow chart of studied population. MOF for Multi-organ failure.

## Abbreviations

MOF: multi-organ failure
ICU: intensive care unit
MAP: mean arterial pressure
OR: odds ratio
SOFA: Sequential Organ Failure Assessment
SAPS II: Simplified Acute Physiologic Score II
HR: heart rate
ROC: receiver operating characteristics
CRT: capillary refill time
