# Supplementary material for: Toe-to-room temperature gradient correlates with tissue perfusion and predicts outcome in selected critically ill patients with severe infections
Source: Ann Intensive Care. 2016 Jul 11;6:63. doi: 10.1186/s13613-016-0164-2 (PMC4940318; doi:10.1186/s13613-016-0164-2)
Supplement: Supplementary file 1 — 10.1186/s13613-016-0164-2 Flow chart of studied population. MOF for Multi-organ failure. [file 13613_2016_164_MOESM1_ESM.pptx]

## Slide 1
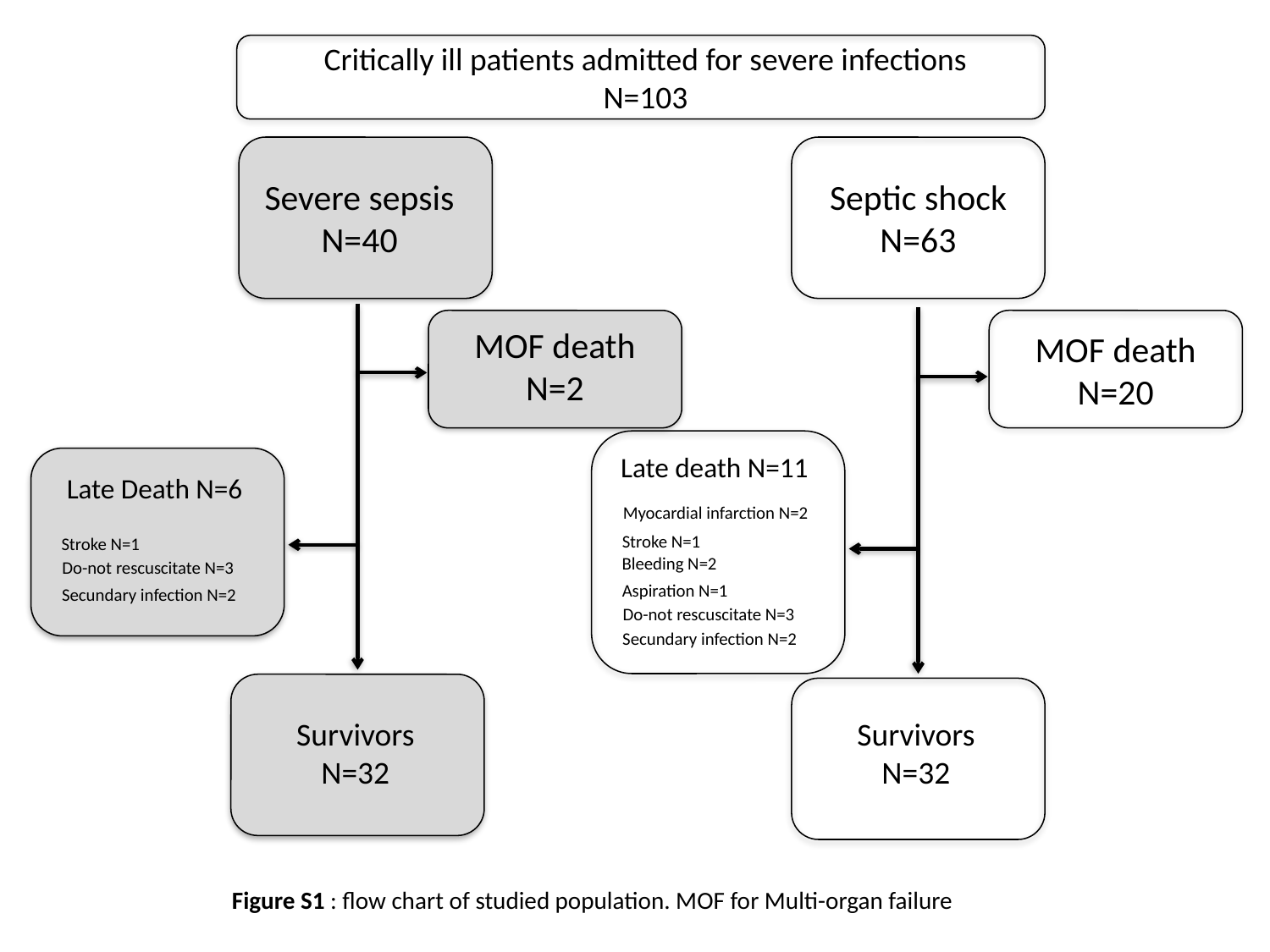

Critically ill patients admitted for severe infections
N=103
Severe sepsis
N=40
Septic shock
N=63
MOF death
N=2
MOF death
N=20
Late death N=11
Late Death N=6
Myocardial infarction N=2
Stroke N=1
Stroke N=1
Bleeding N=2
Do-not rescuscitate N=3
Aspiration N=1
Secundary infection N=2
Do-not rescuscitate N=3
Secundary infection N=2
Survivors
N=32
Survivors
N=32
Figure S1 : flow chart of studied population. MOF for Multi-organ failure
